# Rethinking the Origins of Cross‐Language Effects: How Heard Verbs Influence Japanese‐ and English‐Speaking Children's Attention to the Details of Actions

**DOI:** 10.1111/desc.70138

**Published:** 2026-01-21

**Authors:** Hiromichi Hagihara, Monica Barbir, Hanako Yoshida, Linda B. Smith

**Affiliations:** ^1^ Graduate School of Human Sciences The University of Osaka Suita‐shi Osaka Japan; ^2^ Laboratoire d'Informatique et Systèmes, Institute of Language, Communication and the Brain Aix‐Marseille University Marseille France; ^3^ International Research Center for Neurointelligence (WPI‐IRCN) The University of Tokyo Bunkyo‐ku Tokyo Japan; ^4^ Department of Psychology The University of Houston Houston Texas USA; ^5^ Department of Psychological and Brain Sciences Indiana University Bloomington Indiana USA

**Keywords:** cross‐linguistic comparison, linguistic relativity, verb learning

## Abstract

Languages differ in how words carve up the world into categories, and these differences in lexical categories often influence how speakers interpret perceived events. Past research has shown that languages with a single and general word for one domain tend to cue attention more broadly than languages with multiple, more specific verbs. This supports the idea that the referential range of lexical categories—how broadly or narrowly a word applies—plays a major role in how heard words guide attention and shape interpretations of events. We tested the referential range hypothesis, measuring Japanese‐ and English‐speaking children's (*n* = 236; 24–54 months) interpretations of action events in two conceptual domains: Containment (e.g., putting one object inside another) and Garment‐Closing (e.g., fastening clothing). Japanese lexicalizes containment relations with multiple verbs, whereas English uses one general term. Conversely, English specifies ways of closing garments (e.g., buttoning, zipping, hooking); while Japanese uses a single general verb. Children watched an experimenter demonstrate an action and then selected objects to replicate that action. Across domains and languages, children were tested with Light (e.g., “do”), General (e.g., “close”), or Specific (e.g., “zip”) verbs. The results show that the range of individual lexical categories is not a major determiner of children's interpretations. Verbs with both narrower and broader ranges of use all led to narrow interpretations by children in both languages, but language‐appropriate, atypical specific verbs did not. The full pattern of results raises new hypotheses about cross‐linguistic similarities in verb acquisition and how children learn and interpret verbs.

## 1 Introduction

### 1.1 Words and Their Referents

What are the consequences for cognition if a language has just one lexical category for all the possible kinds of snow, or many distinct lexical categories (Whorf 1956)? What are the consequences for perception if a language collapses blue and green into one lexical category or partitions them into two (Davies 1998; Kay and Cook 2023; Roberson et al. 2004; Winawer et al. 2007)? Does it matter for perception and cognition that English uses two spatial terms (“in” and “on”) to cover a range of relations that Korean carves up differently with five spatial terms (Bowerman and Choi 2001)? The classic debates on the effects of lexical categories on perception and cognition focused on the referential range of lexical categories and were originally framed as an either‐or question (Whorf 1956; Fodor 1983): Does the referential range of the words we use to talk about the world determine how we perceive, represent, and categorize events?

**Summary**

- Japanese‐ and English‐speaking children interpret action verbs in terms of similar narrow categories.
- Light verbs, general verbs, and specific verbs all guide attention to specific actions.

An extensive literature has addressed these questions by studying the perceptual and attentional effects of languages that differ in lexical categories for domains such as color (Daoutis et al. 2006; Kay and Cook 2023; Roberson et al. 2004; Winawer et al. 2007), space (Levinson 2003), number (Frank et al. 2008; Gordon 2004), time (Boroditsky 2001; Chen 2007), pitch (Dolscheid et al. 2013, Dolscheid et al. 2022), and odor (Floyd et al. 2018; Majid 2021; Majid and Burenhult 2014). The rationale underlying many experiments, what we call the *referential range hypothesis*, is the idea that the granularity of the lexical categories determines the breadth or narrowness of people's perception, attention, and/or interpretation of perceived events. The evidence across these many studies shows strong effects of lexical categories, but also contexts in which no effects are observed—even with respect to the same languages and lexical categories (e.g., Boroditsky 2001; Chen 2007; Gordon 2004; Imai and Gentner 1997; for reviews, see Fedorenko et al. 2024; Imai et al. 2016). The current consensus is that the effects of lexical categories on perceptual and conceptual categories are graded, fluid, and context‐dependent (e.g., Dolscheid et al. 2013, Dolscheid et al. 2022; Fausey and Boroditsky 2011; Forder and Lupyan 2019; Sinkeviciute et al. 2024; Winawer et al. 2007; for reviews, see Casasanto 2016; Lupyan 2012). However, the field still knows very little about how and when these effects emerge in development.

The literature on lexical acquisition in children (across a variety of different languages) shows that the referential range of lexical items—their specificity, distinctiveness, concreteness, or abstractness—influences their acquisition and generalization (e.g., Althaus and Westermann 2016; Hall and Bélanger 2005; Havy and Waxman 2016; LaTourrette and Waxman 2020; Ma et al. 2009; Roy et al. 2015; Tan et al. 2024). A large literature also shows direct effects of heard words on attention and memory—in studies of infants, children, and adults—across methods such as preferential looking, visual search, and category generalization (e.g., Fairchild et al. 2018; Forder and Lupyan 2019; Gervits et al. 2023; Huettig and Altmann 2005; Plunkett et al. 2008; Ronfard et al. 2022; Soto and Humphreys 2007; Wolfe et al. 2011; Yoshida and Smith 2005). To better understand how heard words come to guide children's interpretations of observed events, we formulated a series of hypotheses about how the referential range of heard words might influence children's interpretations. To better understand how such effects might lead to cross‐language differences, we compared the performances of children learning two different languages.

### 1.2 Guiding Hypotheses

**FIGURE 1:**
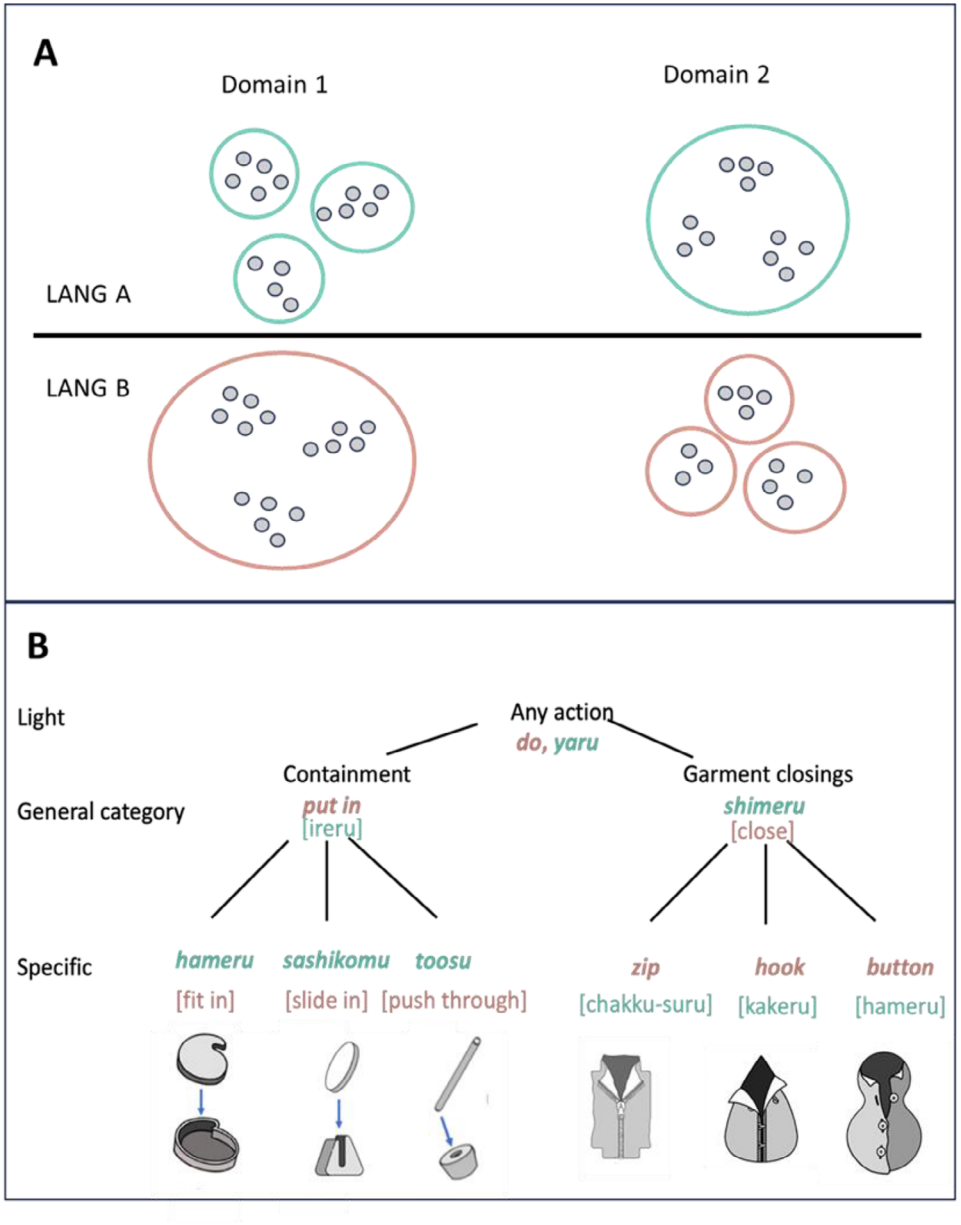
(A) The word‐referent statistical learning hypothesis and (B) English and Japanese lexical categories that provide a test of the hypothesis. (A) Illustration of lexical categories that vary in the range of referents. In Language A (LANG A), lexical categories in Domain 1 have a narrow range and a single lexical category in Domain 2 has a broad range. In Language B (LANG B), the range of lexical categories in the two domains is reversed. (B) Hierarchies of Japanese (in green) and English (in red) verbs differing in their range of referents, with illustrations of exemplar events at the Specific level. The common terms in a language for the action are noted in italics and the less commonly used terms used in the experiment are indicated in brackets.

Figure 1 presents a schematic of how the referential range of lexical categories might differ in two languages. In Language A, Domain 1, a single word refers to a small range of highly similar referents and, as a result, might be expected, to cue a listener to an interpretation in terms of a narrow range of specific instances (Althaus and Plunkett 2016; Althaus and Westermann 2016; Fairchild et al. 2018; Goldstone et al. 2001; Havy and Waxman 2016; LaTourrette and Waxman 2020). In Language B, Domain 1, a single word refers to a broader array of referents (e.g., one word in Language B corresponds to three distinct words in Language A). Under the referential range hypothesis, upon hearing that word, a speaker of Language B would be expected to interpret it as referring to a broad range of possible referents. This is **Hypothesis W (Word)**: *The referential range of the specific heard word determines the narrowness or breadth of interpretation of a perceptual event*.

Many theorists (e.g., Levinson 2003) have focused on the effects of lexical categories at the level of the language as a whole rather than at the level of individual words. Some languages have many verbs and predicates with narrower referential ranges than do other languages. For example, Korean spatial terms have much narrower referential scope than their English counterparts (Bowerman and Choi 2001; Choi 2006). Thus, a key statistical property that biases the range of referents could lie not in individual words but in the overall statistics of the language (Levinson 2003). These ideas suggest **Hypothesis L (language)**: *The referential range of lexical categories in the language influences children's interpretations of a perceptual event, potentially strengthening or weakening the effects of individual heard words*.

Critically, languages may be biased in one direction in one lexical domain but biased in the inverse direction in another domain; that is, within a single language, some domains may run counter to the language‐level statistics. As illustrated in Figure 1A, Language A has many narrow word‐to‐referent mappings relative to Language B in Domain 1, but this pattern is reversed in Domain 2. These observations suggest **Hypothesis D (domain)**: *The effects of heard words on the interpretation of events are strongly organized with respect to conceptual domains*.

Although languages differ in the referential range of lexical categories overall and in specific domains, they also offer multiple ways—at multiple levels of granularity—to refer to the same perceptual event (Butt 2010; Levinson 2003; Maouene et al. 2011; Saji et al. 2011). For example, in LANGUAGE A, one word might typically be used to talk about all events in Domain 2, but the language may also provide other phrases or words that allow speakers to partition those events into smaller categories. Similarly, in LANGUAGE B, speakers may usually use three different words to refer to the three subgroups of these same events, but the language may also offer more general terms that can refer to all events in Domain 2. Under a strong version of Hypothesis W or D, one might predict that the typically used lexical category within its stronger and narrower range would determine the activated concept and thus the interpretation of the perceptual event. Under a weaker hypothesis, the actual heard verb may be the critical determiner and thus interpretations of the same event may vary considerably with the word heard.

Finally, the perceptual properties of the referred to event (e.g., Li and Gleitman 2002) may also play a role and do soespecially for young language learners. For example, although English does not lexically distinguish between putting a key in a lock and putting a cherry in a large bowl, one can easily see the difference. Some studies suggest that infants (Hespos and Spelke 2004) and young learners are biased to more specific or concrete perceptual categories than broader ones (e.g., Göksun et al. 2011; Choi 2006; Imai et al. 2005, 2008; see also Hagihara and Sakagami, 2020; Hagihara et al. 2022). Thus, there is also **Hypothesis PA (perception‐action)**: T*he perceptual properties of the event itself may limit the effects of heard words on the interpretations of those events*.

Languages differ in many interdependent ways. In the study of natural languages’ effects on children's interpretations, there is no way to isolate the different factors raised in the hypotheses in a formal experiment. Instead, the hypotheses served as guides for designing a set of conditions that might allow us to disentangle the relative contributions of the language, the word heard, and the domain in influencing children's interpretations of action events.

### 1.3 Rationale for Selected Languages and Experimental Design

We sought two languages that differed as illustrated in Figure 1A, such that in a dominant Domain (A), one language would lexicalize narrower categories while the other language lexicalized broader ones. In addition, we sought languages for which we could find a second Domain (B) in which the patterns would be reversed. Further, we sought to realize the study using predicates referring to actions because past work shows that languages vary substantially in how they categorize actions (e.g., Frank et al. 2021; Gentner 1982; Gentner and Boroditsky 2001; Levinson 2003; Tardif et al. 2008).

We selected Japanese (LANG A) and English (LANG B) as our case study. Domain 1 consists of actions that create containment relations between a target and a ground object, an event domain that has attracted considerable cross‐linguistic research in children. In English, a ball can be *put in* a box, and a key can be *put in* a lock, even though the physical actions, trajectories of motion, and final relationships between the object and container are quite different. Japanese, in contrast, uses verbs, including *hameru* (fit in), *sashikomu* (slide in, as into a slot), and *toosu* (put through a hole), that carve up the action space into many distinct object‐container relations. Domain 2 consists of actions that involve closing garments. English speakers use specific verbs such as “zipping,” “buckling,” and “buttoning” to refer to specific actions. Japanese speakers typically use a single, more general verb, *shimeru*, to refer to all manners of closing garments, combining the separate lexical categories in English into just one. These two domains—spatial terms and garment closings—are quite different, and we do not see them as symmetrical conditions across the two languages. Spatial terms are relational and inherently abstract; garment closing, in contrast, concerns the state of a single thing—and in English, concerns the specific parts through which the closing of the garment is accomplished. Most studies of cross‐linguistic differences on how lexical categories influence children's interpretation of perceptual events compare contrasting lexical categories with respect to one domain. Using two different domains provides a stronger basis for determining the generality of referential range effects.

These domains also provide a path to determining the role of overall language effects. Linguistic analyses suggest that many Japanese verbs have narrow meanings (Backhouse 1981; Hagihara et al. 2022; Majid et al. 2008; Saji et al. 2024), a property seen in other East Asian languages such as Chinese (Saji et al. 2011). In contrast, many common English verbs often have broader referential ranges (Maouene et al. 2011; Sundquist 2020). Moreover, Japanese speakers often drop the arguments of verbs, which may require listeners to attend to extralinguistic cues for interpretation (Imai et al. 2005, 2008). In contrast, English speakers typically retain arguments— using pronouns or, with young children, the explicit names of things. Do these broader biases, relevant to Hypothesis L, influence interpretations, and do they do so similarly within a language for both domains?

To further disentangle the expected referential range effects, we also measured children's interpretations of observed events in the two domains in the context of other heard verbs. Japanese and English speakers use what are often called light verbs, “*yaru*” and “do,” to refer to a wide range of actions, including containment actions and garment closing actions. Light verbs are often described as having little to no semantic content (Jespersen 1965), as their meaning is derived from co‐occurring words or the extralinguistic context (e.g., “do it”). There are many theoretical debates in linguistics about the standing of light verbs—whether they function as auxiliaries, are delexicalized or semi‐lexicalized, or are polysemous (Brugman 2001; Butt 2010; Butt and Geuder 2001; Mehl 2021), and they may function differently for young language learners than mature ones (e.g., Goldberg et al. 2004; He and Wittenberg 2020; Snedeker and Gleitman 2004; Theakston et al. 2004). However, independent of these issues, both “*yaru*” and “do” are highly frequent in children's input, are produced and comprehended early as generally observed for high‐frequency words (Braginsky et al. 2019; Frank et al. 2021; Sandhofer et al. 2000; Theakston et al. 2004), and are experienced by children in diverse action contexts. Are language and domain effects evident when the heard verb is Light?

Both languages also offer ways to refer to the categories of actions that align with the contrasting lexical categories in the two domains (shown in brackets in Figure 1A). In English, one could ask a child to “close” their coat rather than “zip” it, or to “slide” an object into a slot rather than “put” it in. Likewise, in Japanese, one could use the more specific complex verb *chakku‐suru* rather than *shimeru* to tell a child to zip up their coat, or the more general *ireru* instead of *shashikomu* to tell a child to place a target into a slot. We did not expect these common phrases to be necessarily potent cues, given that they are not the typical words used to talk about these events, but again, they provide insight into how much the heard word—and by comparison how much the language's typical lexical category—determines children's interpretations. In sum, and as illustrated in Figure 1, we use three different verbs to label each event: a light verb, the typically used lexical category, and a language‐appropriate verb at the scale of breadth of the typical lexical category in the other language. For ease of talking about the breadth of the referent range of the three verb conditions, we label them as Light, General, and Specific.

**FIGURE 2:**
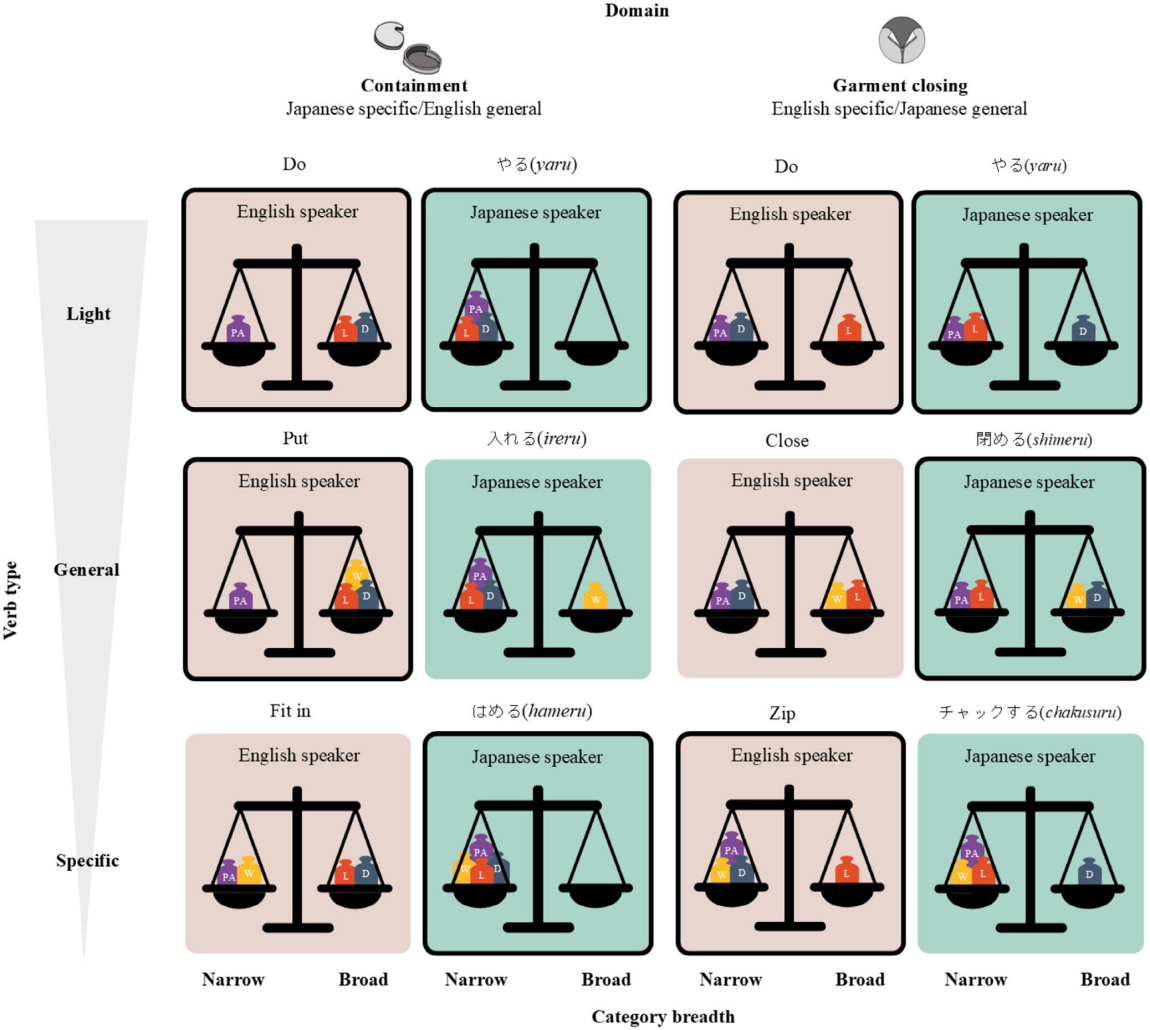
The experimental design and its rationale. The design tracks the potential influence of pertinent verb‐referent statistics at the level of Language (L) in red, Domain (D) in dark blue, Word (W) in yellow, and Perception‐Action (PA) in purple, illustrated as weights on a scale, in the context of Light, General, and Specific verbs that differ in their referential range. Reddish panels indicate expected weights for English‐speaking children, and green panels indicate expected weights for Japanese‐speaking children. Panels with bold outlines indicate the more typical verbs used in each language (see also Figure 1B).

We conceptualized the various verb conditions outlined in Figure 1 as consisting of moving weights on a scale in favor of more or less broad interpretations of an observed event. Thus, the Japanese language biases narrower interpretations of containment events, which could be strengthened by talking about the event with the word *sashikomu* but weakened by hearing the word *ireru*. Figure 2 illustrates our rationale. In the figure, we illustrate the L (language) weights as constant for the language, with English providing an overall bias for broader interpretations and Japanese providing an overall bias for narrower interpretations. We also illustrate the PA (perception‐action) in each domain as a constant weight on the side of narrower interpretations, although it seemed plausible that the PA weight might more strongly push for narrower interpretations of a garment closing event than a containment event. The manipulation of the heard words is illustrated as moving the weights around. Light verbs, for example, could lead to broader interpretations in both languages, or they could activate larger language effects, leading to narrower interpretations in Japanese and broader ones in English. Finally, by requiring attention to the extralinguistic context, they could lead to more detailed interpretations. In Figure 2, we illustrate the light verbs as consistent with language effects (under an assumption that they are so light they have little direct effect) and thus permit language‐consistent broader interpretations for English and narrower ones for Japanese. If the statistics of words in each domain matter, then we would expect the effects to be different for each language across domains. Our question is how the more General and more Specific verbs shift the overall weights in the direction of their referential range and how these might vary with language and domain.

In summary, the motivating goal was to gain insight into how lexical categories such as “put in” versus “*toosu*” that carve up the world into larger versus smaller categories create differences in the interpretation of perceived events and to do so by examining not just these verbs and one domain, but two domains with heard words that might be expected to have broader or narrower or weaker or stronger cuing effects than the commonly used lexical categories in the two languages. We saw the four guiding hypotheses as a first step to a richer understanding of the referential range assumption that has motivated much research in language effects on cognition. Instead, the resulting design—using two languages, two domains, and multiple heard verbs—revealed effects that do not align with any version of the referential range hypotheses.

**FIGURE 3:**
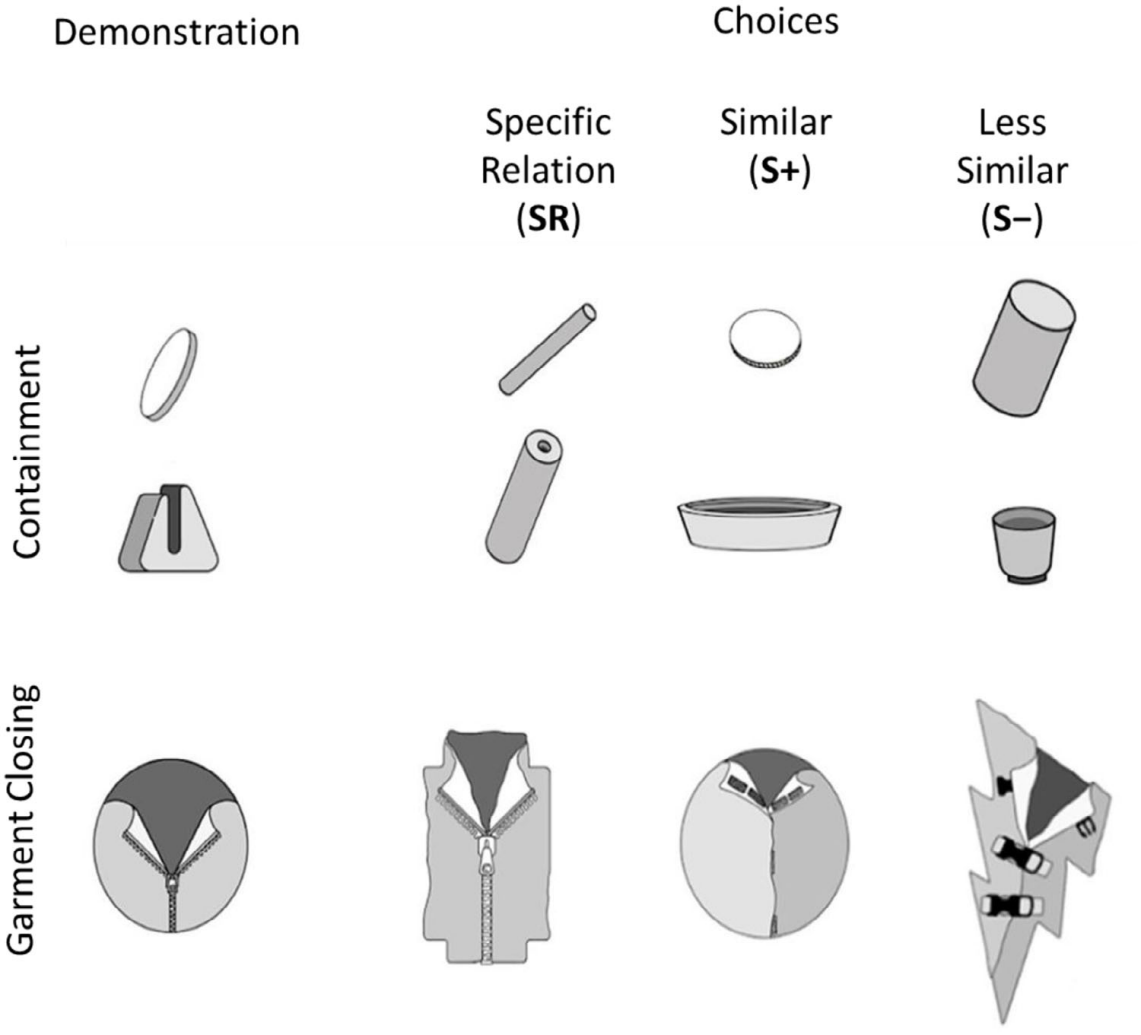
Objects used by the experimenter to demonstrate “sliding in” and “zipping,” along with the choice objects available to the child. In both Containment and Garment‐Closing conditions, the Specific Relation (SR) choice was visually different from the object(s) used in the experimenter's demonstration, but still afforded the specific spatial relation or manner of action lexicalized by the language with the more specific common terms for those actions. The Similar (S+) choice used the same object(s) as in the demonstration, while the Less‐Similar (S‐) choice used object(s) dissimilar to the demonstration. Neither the S+ nor S‐ choices allowed for the same demonstrated specific relation or manner of action.

In the experimental task, children watched the experimenter perform an action, either putting a target into a container or closing a garment. To measure children's interpretation of the action, we presented them with three sets of objects and asked them to select the objects they would use to perform the action demonstrated by the experimenter. The choice of objects constrained the possible action that could be performed. We used children's object selections as the sole dependent measure, as the key question concerned their conceptualization of the observed action rather than their sensory‐motor skills for completing the action. Figure 3 illustrates example trials for the Containment and Garment‐Closing conditions. In the Containment condition, the experimenter had two objects: a target (the disc in the example) and the ground object (the slotted container). The experimenter then stated what she was going to do using the verb assigned to that condition, and subsequently performed the action (e.g., sliding the disc into the slot in the example trial). She then restated what she had done using the same verb. The child was then asked, using the same verb, to perform the action, requiring the child to choose from a set of three alternatives. Critically, none of the alternatives included the exact same objects as those used by the experimenter, preventing exact imitation. Instead, each choice required some form of generalization, differing in the distance and nature of the generalization from the experimenter's demonstration.

In the Containment conditions, the *Specific Relation* (*SR*) choice offered a target and ground object that differed from those used by the experimenter but still created the same SR between the target and ground as would be described by a Specific Japanese verb (*sashikomu* in Figure 3). The *Similar* (*S+*) choice used the same target as the experimenter, but the ground object did not allow the exact same containment relation. However, it did allow a containment relation that would be called “putting in” in English. The *Less‐Similar* (*S‐*) choice used a target and ground object that were both dissimilar to those in the demonstration and did not allow for a spatial relation between target and ground that would be called “putting in.” Instead, it allowed for multiple relations between the two objects including ones that could be called “putting on.”

In the Garment‐Closing conditions, the SR choice presented a garment with a different shape, color, and closing accessory, but one that used the same manner of closing (e.g., zipping). The S+ choice offered a cloth garment identical in color and shape to the one used by the experimenter but with a different closing accessory (e.g., Velcro) that yielded a similar end result (fully closed) to the experimenter's demonstration. The S‐ choice differed from the demonstration garment in color, shape, and accessory and offered a different method of closing with discrete closing points (e.g., buckling). In brief, in both domains, the SR choice was visually different from the object(s) in the observed demonstration but afforded the specific spatial relation or manner of action lexicalized by the language with the more specific common terms for those actions. The other two choices did not allow for the same observed spatial relation or manner of action and differed in their degree of visual and relational similarity to the demonstrated action.

## 2 Method

### 2.1 Participants

A total of 236 children participated in the between‐subjects design: 117 monolingual English‐speaking (E) children, aged 22 to 54 months, living in Bloomington, Indiana, and 119 monolingual Japanese‐speaking (J) children, aged 26 to 53 months, living in Niigata, Japan. Based on previous cross‐linguistic developmental studies (e.g., Choi 2006; Imai et al. 2008; Kuwabara and Smith 2012), we focused on the age at which children's responses might begin to diverge between languages with the goal of detecting early influences on emerging cross‐linguistic differences. Each child participated in one of the three verb conditions: Light verb—*n*(E) = 23; *n*(J) = 23; General verb—*n*(E) = 46; *n*(J) = 49; Specific verb—*n*(E) = 48; *n*(J) = 47. Children from the United States were recruited through outreach events at nursery schools and daycares serving Monroe County, Indiana. Participants broadly represent the demographics of Monroe County, Indiana (approximately 75% European American, 10% African American, 5% Asian American, 5% Latino, and 7% other) and consist predominantly of working‐ and middle‐class families. Children from Japan were recruited through similar outreach efforts and also consist of predominantly working‐ and middle‐class families. Recruitment, consent and data collection were conducted under Protocol #802000161, approved by the Institutional Review Board at Indiana University.

Because we collected data at preschools, many of which had omnibus permission for all children, gender and age information were missing for 25 children (10.6% of the sample), with incomplete demographic data present in both Japanese‐ and English‐speaking groups. Of the children with gender data, 43.6% were girls (English: 47.0%, Japanese: 40.3%). Missing age data were calculated as the mean of the overall sample. A total of four children had missing trial data—two did not choose anything for one trial, and the other two chose two different object sets at the same time. These trials were not included in the analyses, but the four children's data on the remaining trials were included as proportions of completed trials. The experimenter was either fluent in both English and Japanese (author HY) or was a native speaker of the child's language.

### 2.2 Stimuli and Materials

Figure 1B provides the verbs used in each language for the three verb conditions (Light, General, Specific) across the two Domains (Containment, Garment Closing). Figure 3 provides examples of the test trials for the Containment and Garment Closing domains, along with the SR, S+, and S‐ alternative choices. In the 2 (Language) × 2 (Domain) × 3 (Verb) between‐subjects design, each child received only three test trials presented in a fixed order. In the Containment conditions, the order was: tightly fitting, sliding in, and putting through. In the Garment‐Closing conditions, the order was: zip, hook, and button. We chose to test children on just three trials because the goal of the experiment was to determine how child native speakers naturally interpret the heard words. Using just three trials limits the possible effects of children's interpretations being shifted by task demands, being influenced by the experience of different verbs to talk about the same or related actions, and by possible learning across repeated trials. These procedural decisions also enabled us to collect the data in a comparable fashion in nursery school settings despite cultural differences.

### 2.3 Procedure

The experimenter demonstrated an action on an exemplar object set and then prompted the child to choose a test object set. The child chose among the object sets that were placed on a tray with three partitions. The location of the three choices varied across the three test trials for each child. In the English Light verb condition for both Domains (Containment/Garment Closing), the experimenter said “Look. I do this. Can you do it?” For Japanese‐speaking children, the experimenter said, “ほら、お姉さんはこうするよ。*X*ちゃんもこうやってできるかな？” [Hora, onesan wa kou suru yo. *X*‐chan mo kou yatte dekiru kana?], where *X* is the name of the child. For the General and Specific verb conditions, two carrier phrases were used. We did this because English rarely drops the direct object (Sethuraman and Smith 2010) and because novel objects are typically named when introduced (Chen et al. 2021). Accordingly, one carrier phrase used pronouns, while the other used a pseudo‐name for the target object or garment. In the non‐naming version of the Containment Specific verb condition, the experimenter said: “Look. See this. I *V* this. Get yours and *V* it,” where *V* represents the assigned verb. For example, “Look. See this. I slide it in here. Get yours and slide it in.” In the named target version, the experimenter said: “Look. See this *N*. I *V* it. Get your *N* and *V* it,” where *N* is a trial‐specific pseudo noun (*mobit*, *toma*, *zibi*) and *V* is the assigned verb. For example, “Look. See this *mobit*. I slide it in. Get your *mobit* and slide it in.” Japanese carrier phrases followed the same logic. For the Containment condition, the non‐naming carrier phrase was: “ほら、お姉さんは、これをここに*V*よ。*X*ちゃんもこうやってそこに*V*かな？” [Hora, onesan wa kore o koko ni *V* yo. *X*‐chan mo kouyatte soko ni *V* kana?], where *V* is a verb and *X* is the name of the child. The naming carrier phrase was: “ほら、お姉さんの*N*をここに*V*よ。*X*ちゃんの*N*もここに*V*かな？” [Hora, onesan no *N* o koko ni *V* yo. *X*‐chan no *N* mo koko ni *V* kana?], where *N* is a pseudo‐noun (e.g., モビット [mobitto], トマ [toma], and ジビ [zibi]), *V* is a verb, and *X* is the name of the child. Carrier phrases for the Garment‐Closing domain were structured the same way. In all six conditions in both languages, the carrier phrases were semantically and syntactically correct. Task performance was measured by children's choice of the SR objects, and results did not reliably differ between the presence or absence of pseudo‐nouns in the carrier phrase. Thus, we do not discuss the carrier phrases further. The results broken down by carrier phrase are shown in Figure S1.

Object selections were scored as SR, S+, or S‐. Although all children (except the four who did not complete one trial) performed an action on every trial using their selected objects, and all children who selected the SR object attempted to achieve the specific action, scoring was based solely on the objects selected to perform the act and not on the child's speed or complete success in performing the action. Many of these actions (e.g., buttoning, getting a tight‐fit object to go in) require fine motor skills still emerging during this developmental period (Frankenburg and Dodds 1967). The procedure was intuitive and engaging across ages: of the 708 trials, only four lacked a clear selection among the three alternative choices. Because participants selected one object set from three options, chance performance corresponded to 33%.

### 2.4 Data Analysis

The main analyses used Bayesian generalized linear models using *brms* package (Bürkner 2017, 2018, 2021) with a backend of *CmdStanR* (Gabry and Češnovar 2020), an interface to *Stan* (Stan Development Team 2021). Analyses were conducted in *R* (R Core Team 2021) and *RStudio* (RStudio Team 2022). The proportion of SR selections was used as the dependent variable. Independent variables were children's language (English or Japanese), the predicate used (Light, General, or Specific), the domain (Containment or Garment Closing), and their interactions. Children's age (standardized) and pseudo‐noun presence/absence were also included as covariates. The model was fitted using a binomial distribution with a logit link function, which is suitable for regression analysis predicting proportions of correct responses (Gelman et al. 2013; Warton and Hui 2011). For the parameter estimates, uniform distributions were used as priors. We set four chains with an iteration of 12,000 in addition to burn‐in samples of 2000. We confirmed the convergence of parameter estimates following the rules of thumb that Rhat values should be less than 1.1 (Gelman et al. 2013). For the model interpretation, we used the observed means and reported the posterior median (MED) and 95% Bayesian credible intervals (CIs) for parameter estimates (log‐odds scale) or expected values (probability scale). All code and data are available at: https://doi.org/10.17605/OSF.IO/V7U2C.

## 3 Results

**FIGURE 4:**
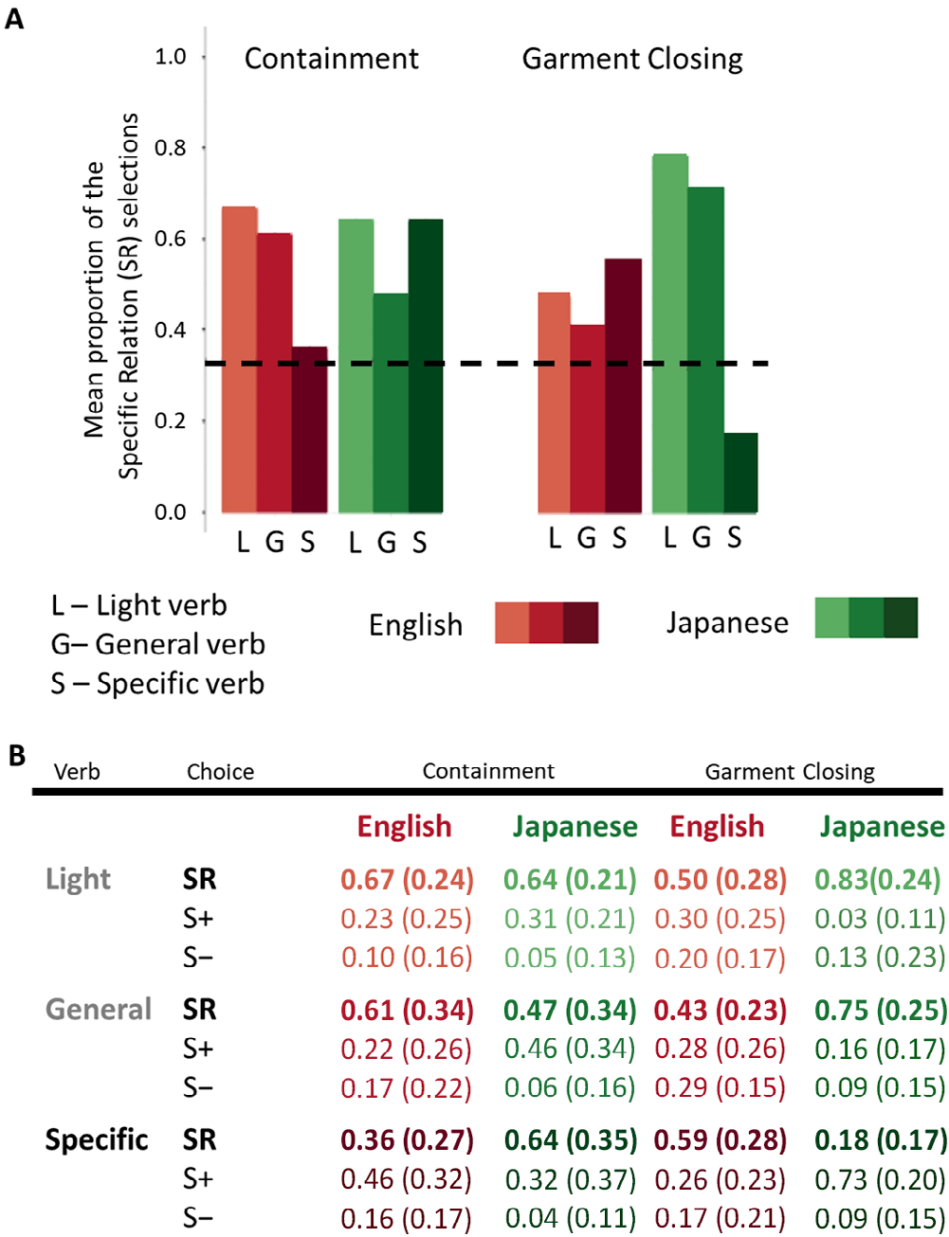
The observed pattern of the Specific‐Relation (SR) selections. (A) The observed pattern of Specific‐Relation choices. The dashed line represents the chance level (0.33), corresponding to random choice among three alternatives. (B) The full pattern of results: mean proportions of SR, Similar (S+), and Less‐Similar (S‐) choices and the standard deviations across the 12 conditions (2 Languages, 2 Domains, 3 levels of Verbs).

If the referential range of the individual heard verb were the sole factor determining the specificity of a child's object selection, we would expect the greatest specificity for Specific verbs, followed by General verbs, and then Light verbs. However, as evident in Figure 4A, this pattern was not observed. A simple version of the referential range hypothesis—as a direct cueing mechanism—is not supported. A Bayesian generalized linear model (parameter estimates and expected values are available in Tables S1 and S2) revealed a main effect of the verb condition, two two‐way interactions (children's language × verb condition and the domain × verb condition), and a three‐way interaction (language × domain × verb condition). The model also revealed an effect of age (parameter estimate, MED = 0.40, CI [0.21, 0.59]), with older children making SR selections more frequently than younger children. These results provide a first indication that language and the words children hear influence their interpretations. However, they also indicate that the factors that influence those interpretations are more complicated than the referential range of the individual words. To better understand these complications, we next consider the Light verb conditions and then the effects of General and Specific verbs on the Containment domainand separately on the Garment‐Closing domain.

### 3.1 Light Verbs

Overall, Light verbs provided good guidance for directing attention to the specific property, though with some variability (Figure 4). A Bayesian generalized linear model revealed that in the Garment‐Closing domain, Japanese‐speaking children chose the SR objects more often than did English‐speaking children (difference in expected value: MED = 0.32, CI [0.08, 0.53]; see also Table S3 for the full pairwise comparisons). SR selections were reliably above chance (0.33) for English‐speaking children in the Containment (expected value: MED = 0.59, CI [0.42, 0.75]) but not in the Garment‐Closing domain (MED = 0.51, CI[0.32, 0.69]). In contrast, Japanese‐speaking children's performance exceeded chance levels in both domains (Containment: MED = 0.62, CI [0.46, 0.77]; Garment Closing: MED = 0.83, CI [0.66, 0.94]). As is apparent in Figure 4B, Japanese‐speaking children were overall more inclined to select the SR objects demonstrated by the experimenter when prompted with a Light verb. In contrast, English‐speaking children were somewhat less likely to do so in the Garment‐Closing domain, where actions are commonly referred to by highly specific verbs. This pattern hints at overall language effects: Japanese verbs tend to be specific, and Japanese children in this study selected the SR objects when given a Light verb.

### 3.2 Containment—General and Specific Verbs

The results in the Containment conditions reveal clear effects of heard words, but nuanced ones that are not a simple consequence of the referential range of individual words. Selection of SR objects was most frequent when the experimenter used the verb most typically used for that action in the child's language—whether general (English “putting in”) or specific (Japanese “*hameru*,” “*sashikomu*,” “*toosu*”). English‐speaking children were less likely to select the objects needed to perform the specific action when the demonstration was labeled with more specific verbs that described the specific action (i.e., “fit in,” “slide in,” or “push through”). As apparent in Figure 4A, English‐speaking children chose SR objects more frequently when cued with the common General verb than with the less commonly used Specific verbs (difference in expected values: MED = 0.29, CI [0.13, 0.44]). When given more specific verbal descriptions of the actions, English‐speaking children tended to choose the S+ objects, which allowed for a putting‐in action but not the specific one demonstrated by the experimenter. For Japanese‐speaking children, the General and Specific verb conditions did not differ statistically, and both led to the selection of the SR objects. Thus, English‐speaking children systematically selected the SR objects more often than chance in the General verb condition (expected value: MED = 0.56, CI [0.43, 0.68]) but not in the Specific verb condition (MED = 0.26, CI [0.17, 0.37]). In contrast, Japanese‐speaking children selected the SR objects above chance levels in both the General verb (MED = 0.50, CI [0.39, 0.61]) and Specific verb (MED = 0.64, CI [0.53, 0.74]) conditions.

### 3.3 Garment Closing—General and Specific Verbs

In the Garment‐Closing domain, English‐speaking children, when given either the Specific and commonly used verb in English or the less common (for garments) General verb (“close”), showed the same pattern as the Japanese‐speaking children in the Containment condition (3.2)—selecting the SR choice, which allowed for the same specific manner of closing the garment. Japanese‐speaking children showed a pattern similar to that observed for English‐speaking children in the Containment condition. That is, Japanese‐speaking children chose the SR object necessary to mimic the specific manner of closing demonstrated by the experimenter when prompted with the General and commonly used verb for garment closing (*shimeru*). However, when prompted with more Specific verbs that distinguished among the three different specific garment‐closing actions, they instead chose the S+ option, which afforded a different manner of closing than the one demonstrated. Japanese‐speaking children chose the SR object more frequently when cued with the General verb than with the Specific verb (difference in expected values: MED = 0.59, CI [0.44, 0.72]). Their SR selections were above chance in the General verb condition (MED = 0.78, CI [0.68, 0.87]) but below chance in the Specific verb condition (MED = 0.19, CI [0.11, 0.30]). In contrast, English‐speaking children's choices did not statistically differ between the verb conditions. They selected the SR object more often than chance in both the General verb (expected value: MED = 0.48, CI [0.35, 0.60]) and Specific verb (MED = 0.61, CI [0.49, 0.73]) conditions.

In brief, the overall pattern in the Garment domain mirrors that of the Containment domain: Commonly used verbs for the action—whether Specific or General—support the selection of SR objects, which are required to perform the same specific action demonstrated by the experimenter. General verbs—whether or not they are the commonly used words in the domain—also lead to more SR selections. However, Specific verbs that accurately describe the SR in the demonstration but are uncommon to the domain in the child's language reduce the frequency of SR selections. Children did not select randomly in this condition; instead, they chose the S+ objects, which allowed for a similar to but not the same action as the SR choice.

In summary, the overall pattern of results shows clear effects of heard words and their status as Light, General, or Specific verbs. There are reliable effects of using different verbs within a language, as well as cross‐language effects. Multiple factors—the referential range of the words, the commonality of their use with respect to the observed action, the language, and the domains—all appear to matter. However, when considering the overall pattern of the data with respect to our analogy of placing weights on different sides of scales (Figure 2), the effect is not simply a matter of adding up the weights on one side or the other. Moreover, the most potent influential factor appears to be the specific relational properties of the observed actions rather than the words heard: Overall, children preferentially select choice objects that are visually very different from the ones used in the demonstration but that allow for creating the same SR.

### 3.4 Patterns of Consistency

**FIGURE 5:**
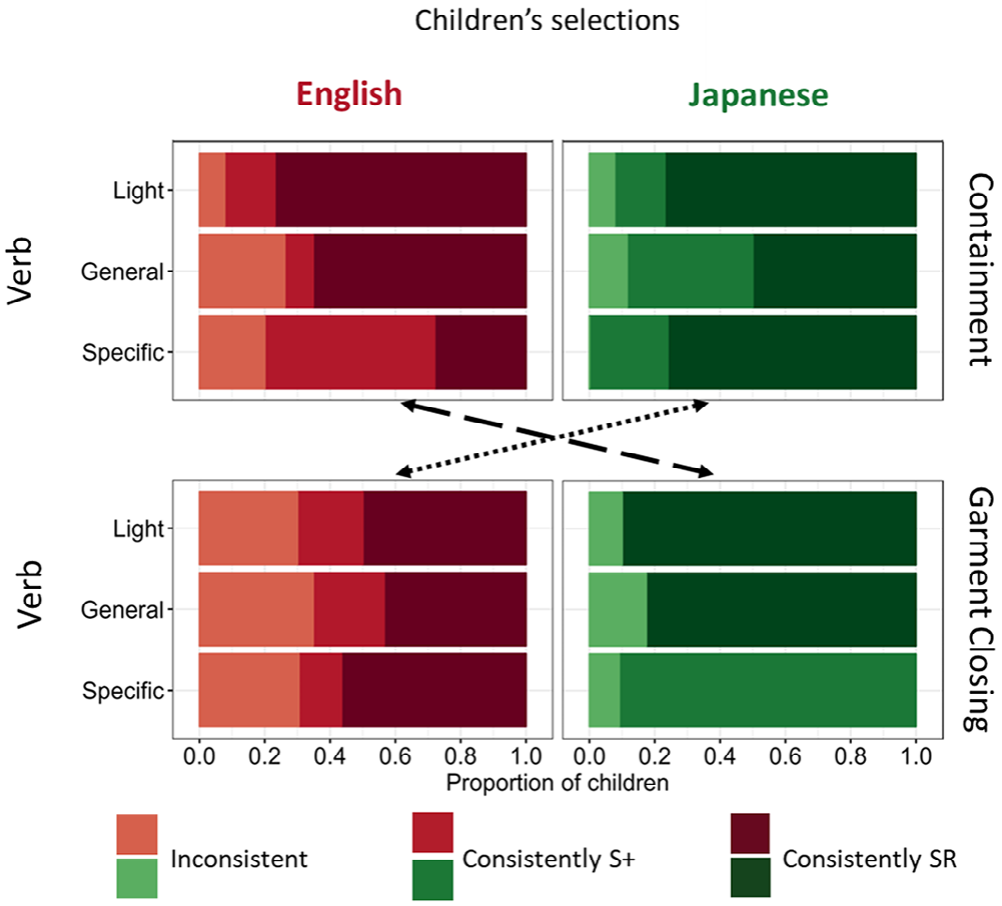
The proportion of English‐ and Japanese‐speaking children in each verb by domain condition whose selections were Inconsistent, Consistently S+, and Consistently SR. The dashed line connects the domains in which the General verb is the commonly used one for each language. The dotted and dashed lines connect the domains in which Specific verbs are the commonly used lexical categories across the two languages.

In the next set of analyses, we used the consistency of individual children's responses as an index of the relative strength of the verbs for cuing the SR. Each participant's object selections were grouped into three categories: *Consistently SR*, if they chose the SR objects on at least 2 of the 3 test trials; *Consistently S+*, if they chose the S+ object on at least 2 of the 3 trials. Any other pattern of response was classified as *Inconsistent*. Figure 5 shows the proportions of children in each condition falling into each category. Collapsing across all conditions and domains, the proportion of children who consistently selected the SR objects did not differ between Japanese‐ and English‐speaking groups (χ^2^(1) = 1.07, *p* = 0.30). This indicates no overall language difference in the bias to copy the SR (with different objects) in the two languages.

To better understand the shared patterns across the two groups of children, the next analyses collapsed across the two domains of Spatial relations and Garment closing, comparing the child consistency patterns for Light, General‐Common (to the domain), General‐Not‐Common (to the domain), Specific‐Common (to the domain), and Specific‐Not‐Common (to the domain) verbs. All the post‐hoc comparisons were pairwise 2 × 2 Chi‐Square contingency tests with Yates correction for continuity. The Type 1 error level was pre‐set at 0.01. The results from the Bayesian generalized linear model were similar and are available in the Supporting Information.

Light verbs served as strong cues for selecting SR objects: 65.2% of English‐speaking children and 82.6% of Japanese‐speaking children consistently chose the SR objects. We compared the numbers of children consistently selecting SR objects when cued with a Light verb versus the verb most commonly used in their language for that domain (for English‐speaking children: the General verb in the Containment domain but the Specific verbs in the Garment‐Closing domain; for Japanese‐speaking children: the Specific verb in the Containment domain but the General verb in the Garment‐Closing domain). We found that Light verbs did not differ from the typically used verbs in the number of children consistently selecting SR objects to imitate the experimenter's actions (χ^2^(1) = 0.07, *p* = 0.79). For children in both language groups, Light verbs directed them toward an interpretation of the observed event that was specific to the performed action.

We next asked whether the most common verb in a domain—collapsing across domains—was more potent than the less commonly used verb. The commonly used verbs—General or Specific—resulted in a greater proportion of children consistently selecting SR objects (70.2% across both languages), compared to the less commonly used verbs (31.2% across both languages) (χ^2^(1) = 27.31, *p* < 0.001). This result indicates a clear effect of the typically used lexical categories on children's interpretation of the experimenter‐demonstrated action of the specific action, regardless of the referential range of the typical verb.

We next considered just the commonly used verbs, again collapsing across domains, and asked whether there were differences in the cueing effects of the Specific verbs (“zip,” “hook,” and “button” in English, “*hameru*,” “*sashikomu*,” and “*toosu*” in Japanese) versus General verbs (“put” in English and “*shimeru*” in Japanese) in the number of children consistently selecting the specific action. This comparison dominates the literature on whether lexical categories in a language determine perceptual and cognitive categories (e.g., Bowerman and Choi 2001; Daoutis et al. 2006; Dolscheid et al. 2013, Dolscheid et al. 2022; Fedorenko et al. 2024; Winawer et al. 2007; Gordon 2004; Kay and Cook 2023; Levinson 2003; Majid and Burenhult 2014; Roberson et al. 2004; Winawer et al. 2007). The overall difference between lexical categories that carve up domains into multiple versus just a single category was not reliable: 66.7% of children cued with a typically‐used Specific verb and 73.9% of children cued with a typically‐used General verb consistently selected the SR objects when imitating the observed action (χ^2^(1) = 0.29, *p* = 0.58). In brief, there is little evidence for a role of the referential range of an individual word in the breadth or narrowness of the interpretation of the demonstrated event.

Finally, we considered the uncommonly used verbs, asking whether there were differences between the cueing effects of more General versus more Specific verbs when they were atypical to the domain. The uncommon‐to‐the domain General verbs (e.g., “*ireru*” in Japanese and “close” in English) led to reliably more children (46.9%) consistently selecting SR objects compared to uncommon‐to‐the domain Specific verbs (e.g., “fit in” in English and “*chakku‐suru*” in Japanese); overall only 14.9% of children across both languages consistently selected the SR objects in this condition (χ^2^(1) = 10.02, *p* = 0.0015). Languages may offer ways to talk about finer distinctions when lexical categories for a domain span multiple perceptible categories, but those more precise phrases did not cue either English‐speaking or Japanese‐speaking children to those narrower perceptual categories. Instead, they pushed children away from that interpretation. As is apparent in Figure 5, this effect is more robust for Japanese‐speaking than English‐speaking children. Critically, it is not that the uncommon specific verbs led to overall inconsistent patterns of selections. Instead, when children were prompted with a more precise but atypical characterization of the observed containment action—such as “fit in” for containment or “*chakku‐suru*” for garment closing—70.2% of the children consistently chose the S+ objects. These objects afforded a different but similar action to the one demonstrated by the experimenter, and one that would be included in the same general lexical category in the child's language, typically captured by a General verb.

In sum, there are overarching similarities in how Light, General, and Specific verbs influence Japanese‐ and English‐speaking children's interpretations of demonstrated actions. However, the overall pattern does not provide strong evidence for the referential range hypothesis at the level of individual words. At best, it hints that a language like Japanese—characterized by more frequent use of specific verb categories—might show a stronger bias for interpretation of the demonstrated action in terms of the specific actions.

## 4 General Discussion

### 4.1 Contributions

The results suggest that lexical categories do not affect our on‐line interpretations in ways strongly affected by the referential range of the individual words, at least not for young language learners. Consistent with prior research (e.g., Hespos and Spelke 2004), children are highly attentive to the specific properties of both containment and garment closing actions, but also appear to encode those events as relational in the sense of being independent to the specific objects used in the demonstration. Embedded in our design was the comparison that dominates studies of lexical category effects on cognition: comparing a language with multiple lexical categories to a language with just one lexical category for a given domain. The present study made two such comparisons: multiple lexical categories for Containment in Japanese versus a single category in English, and multiple lexical categories for Garment Closing in English versus a single category in Japanese. Despite this contrast, there were minimal differences between Japanese‐ and English‐speaking children across both domains. We also examined other verbs in each language. The strength of Light verbs in guiding specific relational representations was greater than that of more specific (but less common) verbs within a domain for speakers of both languages, suggesting perhaps similarities in the ways that children initially interpret Light verbs, a point we will come back to. Finally, the results suggest slightly stronger overall effects among Japanese‐speaking children—a language that has been characterized as having verbs with more concrete meanings (Allen and Conklin 2014, see also for related evidence from Korean, Choi and Gopnik 1995)—compared to English. The whole pattern cannot be readily explained by the referential range hypothesis that has been the foundation for much previous research. Overall, the results indicate that there is much that the field does not know about children's early understanding of verbs, nor the developmental emergence of cross‐linguistic effects on perception and cognition.

### 4.2 Beyond Lexical Categories

**FIGURE 6:**
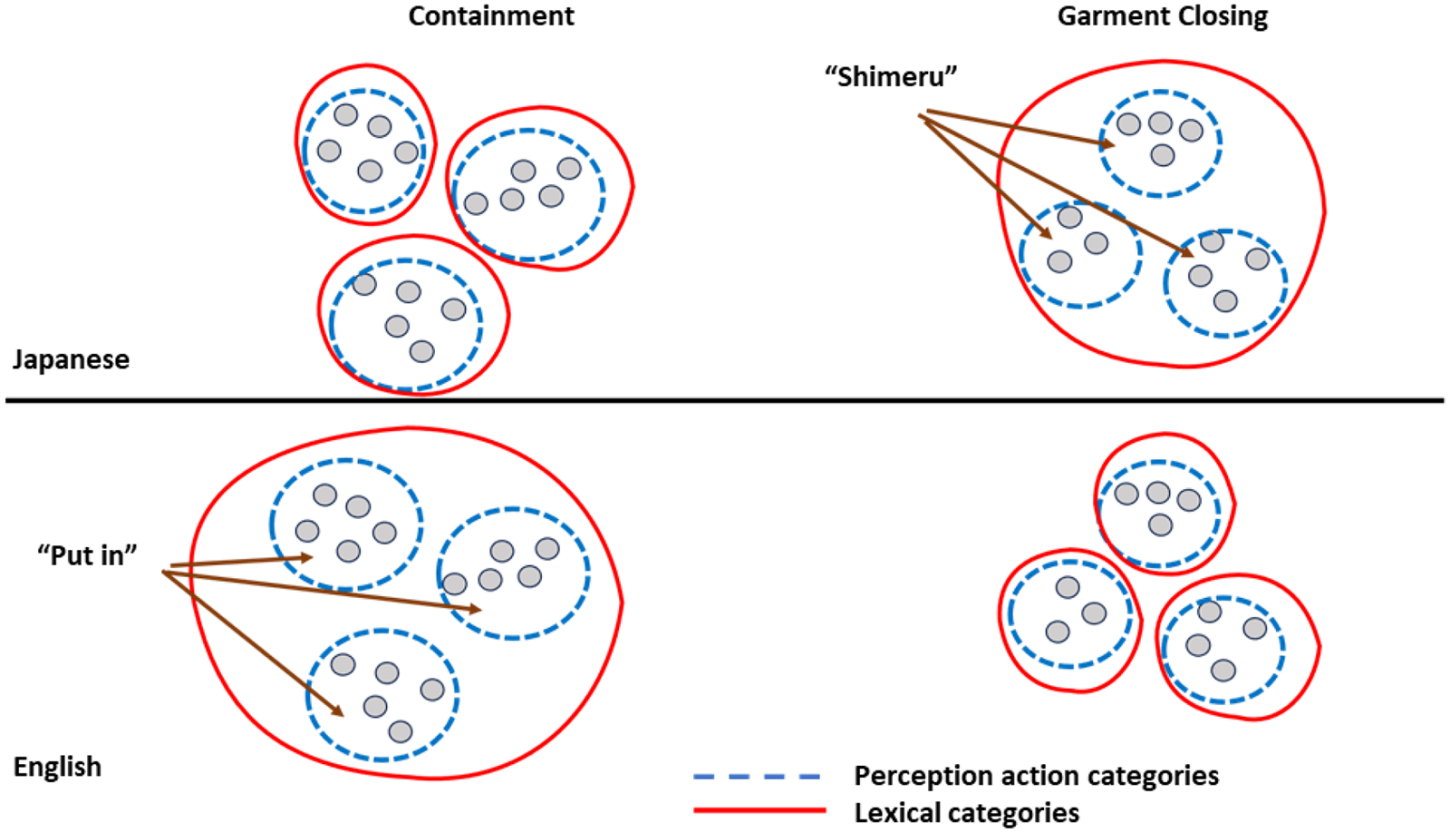
An illustration of the idea that children in each language have pre‐formed and similar sensory‐motor categories to which lexical categories are mapped.

Figure 6 offers a revised version of Figure 1. The starting point for learning the verbs examined in this study may be the PA categories. Very young children spend a great deal of time acting on objects—putting things in other things, closing garments. These already‐formed categories may change little with the learning of words. Rather, the words may bend toward the categories. For young English‐speaking children, “put in” may not be represented in terms of the shared properties across acts of fitting in, sliding in, and pushing through. Instead, “putting in” may be represented as a conjunction of subcategories, where “putting in” means “fitting in” *or* “sliding in” *or* “pushing through.” Likewise, *shimeru* may not mean “close” to Japanese‐speaking children in the sense of shared properties across closing doors, clothes, and boxes. Instead, in the context of clothing, it may function as a conjunction of separate action categories, such as “buttoning,” “hooking,” or “zipping,” This possibility is reminiscent of linguistic arguments suggesting that some broadly used verbs may be better understood not as highly abstract terms, but as polysemous (Brugman 2001). Additionally, children's interpretations of the light verbs “do” and *yaru* may be strongly informed by the underlying perceptual categories because these verbs are inherently context‐dependent and thus direct attention to the extralinguistic context and the already‐formed PA categories. In sum, the pattern of results indicates that verb learning may not begin with the heard words but is instead built upon prior experiences in perception and action.

However, attention to the details of perceptual events, regardless of the heard word, is not sufficient to explain the findings. The PA events in children's imitations maintained the abstract action relevant to either the verbs in their own language or the verbs in the other language. Where does this one‐level of initial abstraction come from? One possibility is that it comes from the syntactic frames in which the heard verb is embedded. Tomasello's “verb‐island” hypothesis (Tomasello 1992, 2003; see also Gleitman 1990; Goldberg 1995) proposes that verb meanings developmentally emerge from their links to specific syntactic frames. The sentence frames surrounding the verb in the present task—rather than the heard verb—may play a role in directing attention to the relational aspects of actions rather than to the specific but irrelevant properties of acted‐on objects involved, such as the shape of the object that is put in a container or the color, shape, and details of the garment. If this is so, syntactic frames may play a critical role in the emergence of cross‐language effects of heard verbs on interpretations of events (Fausey and Boroditsky 2011).

If children have strong and well‐formed PA categories, why does a less typical but linguistically appropriate and specific description of an action—albeit uncommon— shift attention away from the objects needed to perform the observed event? We offer two hypotheses worthy of empirical study. First, competitive processes in lexical learning, comprehension, and production are well‐documented across all levels of human language (Dell and O'Seaghdha 1992; MacWhinney 1987; Stella et al. 2024). The straightforward prediction is this: To the degree that observing an object being tightly fit into a container activates “putting in,” the interpretation of different words (e.g., “fitting in”) may compete with and shift away from this observed action. This competitive effect is well‐documented in children in pragmatic phenomena (Ninio and Snow 1996; Bohn et al. 2021) as well as in word learning and word‐referent judgements (Merriman et al. 1989; Wu et al. 2022; Lewis et al. 2020; Bohn et al. 2021). However, these competitive effects were not observed for Light verbs or for uncommon General terms within a domain (“close” in English, *ireru* in Japanese). A second hypothesis is that the surrounding syntactic frames are making important contributions to children's interpretation of the action event. The less common General terms have sentence frames similar to those used in the Light and Common verb (both General and Specific) conditions. However, the less common Specific verbs sometimes did not. The role of syntactic frames is a critical target for future study.

### 4.3 Can We Conclude That the Referential Range Hypothesis Is Wrong?

One possibility is that the specific words and testing context used in the present study are unique and the referential range hypothesis applies for many other lexical categories. The PA categories for containment and garment closing may be so strong that they are, at best, minimally affected by lexicalization. That is, zipping versus hooking or fitting an object tightly into a receptacle versus through an opening, may be so distinct that they may never form a unitary abstract category, even in adults. Language effects on cognition are known to be context‐dependent, nuanced, not all‐or‐none, and may well be more pronounced for certain kinds of concepts than for others (e.g., Chen et al. 2015; Imai and Mazuka 2007; Landau et al. 2023; Papafragou et al. 2008).

Alternatively, stronger language effects might be detected using different methods to test young children. The present task, which required children to plan an action, is arguably a context that might have diminished the effects of the heard words. If this hypothesis is correct, the prediction is that adult speakers would perform similarly to children in the present task. This hypothesis leaves open the possibility of clear support for the referential range hypothesis if the task used more symbolic and less well‐differentiated stimuli such as static black‐and‐white photos of the end result of actions. This conjecture is consistent with several studies indicating that cross‐linguistic effects are less likely to be found in real‐world contexts than in pictures (Chen et al. 2015; Landau et al. 2023; Papafragou et al. 2008; Regier and Zheng 2007). If this hypothesis is generally correct—that action verbs activate different *meanings* in the context of planning an action (“put it in”) versus visually recognizing a scene (“which picture shows” “putting in”)—it suggests that the same word may engage systematically different senses depending on the activated neural networks (Carota et al. 2023; Yang et al. 2017; Tarhan et al. 2021). Alternatively, this could mean that cross‐linguistic effects are best observed in ambiguous or unclear contexts, where heard words serve as the primary cue for concept activation.

Finally, cross‐linguistic effects—even with respect to Containment and Garment‐Closing predicates in an action‐performing context—may emerge later in development, as language learning becomes more advanced (Aussems et al. 2022). This hypothesis is consistent with arguments that early word meanings are fundamentally different from mature ones (Saji et al. 2011; Ambridge et al. 2013; Hagihara and Sakagami, 2020; Hagihara et al. 2022; Hagihara 2024). There are both findings and hypotheses in the literature that the statistics of syntactic frames (e.g., active, passive, transitive, intransitive), rather than verb‐referent statistics, play the key role in the development of more abstract verb meanings and cross‐linguistic differences (e.g., Ambridge et al. 2013; Scott and Fisher 2009; Sethuraman et al. 2011). Thus, the emergence of cross‐linguistic effects may involve more than the perceptual‐action categories and the lexical categories, the focus of the present study, including composition of the entire early learned lexicon and the syntactic frames in which those words appear (Saffran et al. 1996; Mintz 2003; Barbir et al. 2023).

In conclusion, there is a great deal that the field needs to determine about the developmental pathways and contexts through which the languages people speak influence their interpretations of events in the world. While studies on lexical category effects often show strong influences, they do not always do so. The field needs to move beyond demonstrations of lexical category effects to examining lexical systems, the syntactic frames in which individual words appear, and their role in shaping how the words we hear in a given context come to guide our thoughts.

## Author Contributions

**Hiromichi Hagihara**: conceptualization, formal analysis, data curation, writing – original draft, writing – review and editing, visualization, project administration. **Monica Barbir**: conceptualization, formal analysis, writing – original draft, writing – review and editing, visualization, supervision, project administration. **Hanako Yoshida**: conceptualization, methodology, investigation, resources, data collection, curation, writing – review and editing, supervision, project administration, funding for data collection. **Linda B. Smith**: conceptualization, methodology, investigation, resources, data collection, curation, writing – review and revisions, supervision, project administration, funding for data collection.

## Funding

This research was supported by the World Premier International Research Center Initiative (WPI), MEXT, Japan; Foundation Fyssen postdoctoral study grant (MB); Japan Society for the Promotion of Science post‐doctoral fellowship grant P20722 (MB); Horizon Europe Marie Skłodowska‐Curie Actions Postdoctoral fellowship 101151855 (MB); JSPS KAKENHI Grant Numbers JP 22KJ0525 and JP22K13664 (HH). Data collection was supported by National Institutes of Health R01 MH60200 (LBS) and funds from Indiana University (LBS).

## Ethics Statement

This research was approved by the Institutional Review Board at Indiana University, Protocol number 802000161.

## Conflicts of Interest

The authors declare no conflicts of interest.

## Supporting information




**Supporting File 1**: desc70138‐sup‐0001‐SuppMat.pdf

## Data Availability

All code and data used in this study are available at: https://doi.org/10.17605/OSF.IO/V7U2C.
