# Supplementary material for: Rethinking the Origins of Cross‐Language Effects: How Heard Verbs Influence Japanese‐ and English‐Speaking Children's Attention to the Details of Actions
Source: Dev Sci. 2026 Jan 21;29(2):e70138. doi: 10.1111/desc.70138 (PMC12822245; doi:10.1111/desc.70138)
Supplement: Supplementary file 1 — Supporting File 1: desc70138‐sup‐0001‐SuppMat.pdf [file DESC-29-e70138-s001.pdf]

Supplementary Information

Rethinking the origins of cross-language effects: How heard verbs influence Japanese- and English-speaking children's attention to the details of actions

Figure S1

Proportions of Specific-Relation (SR) selections across conditions, domains, and pseudo-noun presence.

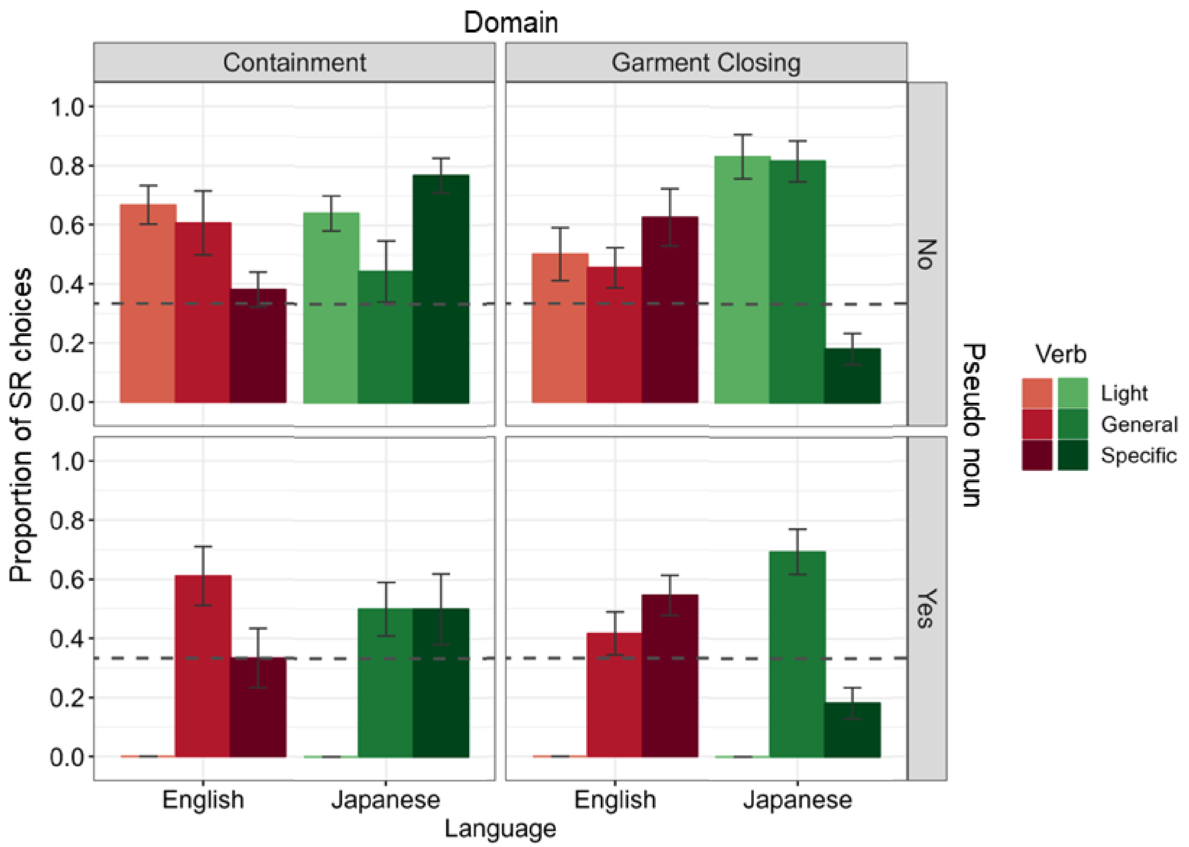

Note. The mean proportions of Specific-Relation (SR) selections. Error bars indicate standard errors. The dashed lines represent the chance level (.33). The task performance did not reliably differ between the presence or absence of the pseudo-names in the carrier phrase. To confirm this, we created models predicting the proportion of SR selections in either General or Specific verb conditions, while fixed effects included children's language, the domain, the pseudo-name presence, and their interactions, as well as children's age as a covariate. For both models, we did not detect any main effects and interactions for the pseudo-names. The only exception was that in Japanese children in the Containment domain with Specific verb condition, the task performance was higher when the pseudo-name was absent than when it was present (difference in expected values, MED = .27, CI[.06, .47]).

Table S1

Parameter estimates for the primary analysis

Parameter	Posterior median	95% CI
Intercept	0.53	[-0.14, 1.24]
Language (Japanese)	0.14	[-0.82, 1.09]
Domain (Garment Closing)	-0.34	[-1.35, 0.68]
Verb (General)	-0.13	[-1.02, 0.73]
**Verb (Specific)**	**-1.40**	**[-2.29, -0.55]**
Language × Domain	1.43	[-0.11, 3.06]
Language × Verb (General)	-0.37	[-1.53, 0.80]
**Language × Verb (Specific)**	**1.48**	**[0.31, 2.66]**
Domain × Verb (General)	0.01	[-1.20, 1.24]
**Domain × Verb (Specific)**	**1.84**	**[0.62, 3.08]**
Language × Domain × Verb (General)	0.18	[-1.72, 2.00]
**Language × Domain × Verb (Specific)**	**-4.98**	**[-6.90, -3.10]**
**Age in months (standardized)**	**0.40**	**[0.21, 0.59]**
Pseudo-noun presence	-0.34	[-0.70, 0.01]

Note. Parameter estimates are reported on the log-odds scale. English-speaking children in Light verb condition without pseudo-nouns in Containment domain served as the reference in the model. Detected effects are in bold case.

Table S2

Expected values for the proportions of the Specific-Relation (SR) selections

Domain	Language	Verb	Posterior median	95% CI
Containment	English	Light	**.59**	**[.42, .75]**
		General	**.56**	**[.43, .68]**
		Specific	.26	[.17, .37]
	Japanese	Light	**.62**	**[.46, .77]**
		General	**.50**	**[.39, .61]**
		Specific	**.64**	**[.53, .74]**
Garment Closing	English	Light	.51	[.32, .69]
		General	**.48**	**[.35, .60]**
		Specific	**.61**	**[.49, .73]**
	Japanese	Light	**.83**	**[.66, .94]**
		General	**.79**	**[.68, .87]**
		Specific	**.19**	**[.11, .30]**

Note. Children's age and the pseudo-noun presence were averaged. Bold cases indicate the expected values were above or below the chance level (.33).

Table S3

Pairwise comparisons of expected values for the proportions of the Specific Relation (SR) selections

Contrast		Difference	
		Posterior median	95% CI
Light verbs			
English - Containment	Japanese - Containment	–. 03	[–.25, .19]
**English - Garment Closing**	**Japanese - Garment Closing**	**–.32**	**[–.53, –.08]**
English - Containment	English - Garment Closing	.08	[–.16, .32]
Japanese - Containment	Japanese - Garment Closing	–.20	[–.40, .01]
Containment			
English - General verb	Japanese - General verb	.06	[–.11, .22]
**English - Specific verb**	**Japanese - Specific verb**	**–.38**	**[–.52, –.22]**
**English - General verb**	**English - Specific verb**	**.29**	**[.13, .44]**
Japanese - General verb	Japanese - Specific verb	–.14	[–.29, .02]
Garment Closing			
**English - General verb**	**Japanese - General verb**	**–.31**	**[–.46, –.15]**
**English - Specific verb**	**Japanese - Specific verb**	**.42**	**[.26, .56]**
English - General verb	English - Specific verb	–.14	[–.30, .03]
**Japanese - General verb**	**Japanese - Specific verb**	**.59**	**[.44, .72]**

Note. Children's age and the pseudo-noun presence were averaged. Detected effects are in bold case.

Supplementary Analysis:

Patterns of consistency using a Bayesian generalized linear model

A Bayesian generalized linear model was performed with the proportion of children who were classified as *Consistently Specific-Relations* (SR) as the dependent variable. The independent variables included children's language (English or Japanese), verb used (Light, Common, or Uncommon), and their interactions, with covariates of children's age in months (standardized) and pseudo-noun presence. Other specifications are the same as in the main analysis.

Overall results are shown in Table S4. As seen in the parameter estimates in this table, the main effects of language (MED = 1.11, CI[0.18, 2.10]) and uncommon verbs (MED = –1.27, CI[–2.18, -0.38]) were detected. No interactions were found. This suggests that the proportion of children who consistently chose the SR options was greater in Japanese-speaking than in English-speaking children, and when common verbs were used than when uncommon verbs were used. Compatible with the main analysis, older children were more likely to be categorized as Consistently SR than younger children (MED = 0.57, CI[0.27, 0.90]). In the following, we focused on the differences in expected values among conditions while controlling the covariates of children's age and pseudo-noun presence.

Consider first Light verbs. When we compared the proportion of children consistently performing SR when cued with a Light verb versus the verb most commonly used in the domain for their language (for English-speaking children, the General verbs in the Containment domain and the Specific verbs in the Garment closing domain; for Japanese-speaking children, the Specific verbs in the Containment domain and the General verbs in the Garment closing domain), Light verbs did not differ from the usually used verbs, providing just as potent a cue to a specific action, for both the English- (difference in expected values, MED = .05, CI[.–

22, .30]) and Japanese-speaking children (MED = .03, CI[–.19, .19). The proportion of children categorized as Consistently SR under Light verbs also exceeded the chance level (7/27 patterns = .26) for both the English- (expected value, MED = .65, CI[.42, .83]) and Japanese-speaking children (MED = .84, CI[.65, .95]).

Table S4

Parameter estimates for the exploratory analysis

Parameter	Posterior median	95% CI
Intercept	0.47	[–0.22, 1.20]
**Language (Japanese)**	**1.11**	**[0.18, 2.10]**
Verb (Light)	0.22	[–0.91, 1.39]
**Verb (Uncommon)**	**–1.27**	**[–2.18, –0.38]**
Language × Verb (Light)	–0.03	[–1.74, 1.73]
Language × Verb (Uncommon)	–1.13	[–2.47, 0.19]
**Age in months (standardized)**	**0.57**	**[0.27, 0.90]**
Pseudo-noun presence	–0.17	[–0.84, 0.49]

Note. English-speaking children in Common Verb condition served as the reference in the model. Detected effects are in bold case.

We next asked whether the most commonly used verb in a domain was potent than less commonly used verb (excluding Light verbs from that category). The commonly used verb – be it general or specific – resulted in more children consistently selecting SR options than uncommon verbs for both the English-speaking (difference in expected values, MED = .30, CI[.09, .49]) and Japanese-speaking children (MED = .52, CI[.34, .68]). Commonly used verbs led to a higher proportion of children categorized as Consistently SR than chance for the English- (expected value, MED = .60, CI[.45, .73]) and Japanese-speaking children (MED = .82, CI[.69, .91]). We then considered just the commonly used verbs and asked whether the

there were differences between the cueing effects of commonly used Specific verbs (*zip*, *hook* and *buckle* in English, *hameru*, *sashikomu*, and *toosu* in Japanese) versus commonly used General verbs (*put in* English and *shimeru* in Japanese) in the number of children consistently selecting the SR choice. Another Bayesian generalized linear model was fit to the subset of the data collapsing the two languages with the fixed effects of verb meaning specificity (Specific or General) while controlling children's age and pseudo-noun presence. No difference between the verb types was detected (parameter estimate, MED = 0.34, CI[–0.61, 1.32]; see Table S5 for the other estimates). Children consistently selected the SR actions more than chance as long as commonly used verbs were given, regardless of their Specific (expected value, MED = .69, CI[.55, .81]) or General semantic breadth (MED = .76, CI[.61, .87]).

Table S5

Parameter estimates for the exploratory analysis (Common verbs only)

Parameter	Posterior median	95% CI
Intercept	1.12	[0.34, 1.97]
Verb (General)	0.34	[–0.61, 1.32]
**Age in months (standardized)**	**0.71**	**[0.15, 1.34]**
Pseudo-noun presence	–0.60	[–1.58, 0.34]

Note. Children in Specific Verb condition served as the reference in the model. Detected effects are in bold case.

We next considered the uncommonly used (in the domain) General and Specific verbs. The proportion of children categorized as Consistently SR under uncommon verbs remained at chance for both the English- (expected value, MED = .29, CI[.17, .44]) and Japanese-speaking children (MED = .29, CI[.17, .44]). To ask whether there were differences between the cuing effects, we performed a Bayesian generalized linear model to the subset of the data consisting

of only uncommon verbs, with children's language collapsed. The uncommon general class verbs (*ireru* in Japanese and *close* in English) led to reliably more children consistently selecting the SR choice than the less commonly-used specific verbs (e.g., *fit it in* in English and *kakeru* in Japanese) (parameter estimate, MED = 3.14, CI[1.73, 4.92]; see Table S6). Although uncommon general verbs led to a greater number of children selecting the SR than chance (expected value, MED = .56, CI[.40, .71]), specific verbs that are not the typical verb used for talking about a specific action, shifted children away from selecting SR options. This led to only 14.9% of children choosing the SR, which was below chance (MED = .05, CI[.01, .15]).

Table S6

Parameter estimates for the exploratory analysis (Uncommon verbs only)

Parameter	Posterior median	95% CI
Intercept	–2.86	[–4.38, –1.67]
**Verb (General)**	**3.14**	**[1.73, 4.92]**
**Age in months (standardized)**	**1.10**	**[0.53, 1.78]**
Pseudo-noun presence	–0.06	[–1.11, 0.96]

Note. Children in Specific Verb condition served as the reference in the model. Detected effects are in bold case.

Table S7

Parameter estimates for the analysis of the age-related change

Parameter	Posterior median	95% CI
**Intercept**	**0.46**	**[0.02, 0.90]**
**Language (Japanese)**	**0.35**	**[0.00, 0.70]**
**Age in months (standardized)**	**0.33**	**[0.11, 0.55]**
Language × Age in months	0.02	[-0.37, 0.42]
Domain (Garment Closing)	0.09	[-0.26, 0.45]
Verb (Common)	-0.01	[-0.48, 0.45]
**Verb (Uncommon)**	**-1.24**	**[-1.70, -0.79]**

Note. English-speaking children in Light verb condition in Containment domain served as the reference in the model. Detected effects are in bold case.

Table S8

Parameter estimates for the analysis of the age-related change (Common verbs condition only)

Parameter	Posterior median	95% CI
Intercept	0.16	[-0.32, 0.64]
**Language (Japanese)**	**0.68**	**[0.10, 1.30]**
**Age in months (standardized)**	**0.41**	**[0.01, 0.82]**
Language × Age in months	-0.05	[-0.70, 0.63]
Domain (Garment Closing)	0.36	[-0.25, 0.98]

Note. English-speaking children in Containment domain served as the reference in the model. Detected effects are in bold case.
